# Two *Pantoea agglomerans* type III effectors can transform nonpathogenic and phytopathogenic bacteria into host‐specific gall‐forming pathogens

**DOI:** 10.1111/mpp.12860

**Published:** 2019-08-01

**Authors:** Gal Nissan, Laura Chalupowicz, Guido Sessa, Shulamit Manulis‐Sasson, Isaac Barash

**Affiliations:** ^1^ School of Plant Sciences and Security, Faculty of Life Sciences Tel‐Aviv University Tel Aviv Israel; ^2^ Department of Plant Pathology and Weed Research ARO the Volcani Center Rishon LeZion 7528809 Israel

**Keywords:** effectors, galls formation, host specificity, host‐specific transcription activators, *Pantoea agglomerans*, type III secretion system

## Abstract

*Pantoea agglomerans* (*Pa*), a widespread commensal bacterium, has evolved into a host‐specific gall‐forming pathogen on gypsophila and beet by acquiring a plasmid harbouring a type III secretion system (T3SS) and effectors (T3Es). *Pantoea agglomerans* pv. *gypsophilae* (*Pag*) elicits galls on gypsophila and a hypersensitive response on beet, whereas *P. agglomerans* pv. *betae* (*Pab*) elicits galls on beet and gypsophila. HsvG and HsvB are two paralogous T3Es present in both pathovars and act as host‐specific transcription activators on gypsophila and beet, respectively. PthG and PseB are major T3Es that contribute to gall development of *Pag* and *Pab*, respectively. To establish the minimal combinations of T3Es that are sufficient to elicit gall symptoms, strains of the nonpathogenic bacteria *Pseudomonas fluorescens* 55, *Pa* 3‐1, *Pa* 98 and *Escherichia coli*, transformed with pHIR11 harbouring a T3SS, and the phytopathogenic bacteria *Erwinia amylovora*, *Dickeya solani* and *Xanthomonas campestris* pv. *campestris* were transformed with the T3Es *hsvG*, *hsvB*, *pthG* and *pseB*, either individually or in pairs, and used to infect gypsophila and beet. Strikingly, all the tested nonpathogenic and phytopathogenic bacterial strains harbouring *hsvG* and *pthG* incited galls on gypsophila, whereas strains harbouring *hsvB* and *pseB*, with the exception of *E. coli*, incited galls on beet.

*Pantoea agglomerans* (*Pa*), a widespread commensal Gram‐negative bacterium, is distributed in many diverse habitats and commonly associated with plants as an epiphyte and/or endophyte (Kobayashi and Palumbo, 2000; Lindow and Brandl, 2003). *Pantoea agglomerans* pv. *gypsophilae* (*Pag*) (formerly known as *Erwinia herbicola* pv. *gypsophilae*) and *P. agglomerans* pv. *betae* (*Pab*) are two related tumorigenic pathovars. *Pag* elicits gall formation on gypsophila and a hypersensitive response (HR) on beet, whereas *Pab* incites galls on beet and gypsophila (Burr *et al.*, 1991; Cooksey, 1986). The virulence of both pathovars relies on the presence of a pathogenicity plasmid (pPATH) containing a pathogenicity island (PAI), which is distributed among genetically diverse populations of *P. agglomerans* (reviewed in Barash and Manulis‐Sasson, 2009). The pathogenicity plasmids of *Pag* and *Pab* may vary in size and their curing results in a loss of pathogenicity (Weinthal *et al.*, 2007). Studies on the pPATH_Pag_ of strain *Pag *824‐1 revealed a plasmid size of approximately 135 kb accommodating a PAI of nearly 75 kb (Barash and Manulis‐Sasson, 2009). The PAI harbours an intact *hrp*/*hrc* (hypersensitive response and pathogenicity/hypersensitive response and conserved) gene. It contains a functional type III secretion system (T3SS), type III effectors (T3Es), multiple and diverse insertion sequences, which presumably were involved in the evolution of the pPATH_Pag_ (Guo *et al.*, 2002), and a cluster of genes encoding biosynthetic enzymes of the plant hormones auxin and cytokinins (Barash and Manulis‐Sasson, 2009). The structure of the PAI, its plasmid location and the observation that all the identified T3Es in *Pab *4188 and *Pag *824‐1 were plasmid‐borne (Nissan *et al.*, 2018) strongly suggest a recent evolution of pathogenesis.

A draft genome of *Pab *4188 and *Pag *824‐1 combined with a machine‐learning approach followed by a translocation assay of T3Es into beet roots and pathogenicity assays were recently employed to reveal the inventories of T3Es in the two pathovars (Nissan *et al.*, 2018). Eight *Pab *4188 functional plasmid‐borne T3Es could trigger galls on beet and gypsophila, whereas nine plasmid‐borne T3Es of *Pag *824‐1 could trigger galls on gypsophila and HR on beet (Nissan *et al.*, 2018). In contrast to the small repertoire of T3Es in *Pab* or *Pag*, pathovars of other phytopathogenic bacteria, including *Pseudomonas syringae* pv. *tomato* (DC3000), *Xanthomonas euvesicatoria* or *Ralstonia solani*, harbour considerably larger pools of about 30 or more T3Es (Genin and Denny, 2012; Kvitko *et al.*, 2009; Teper *et al.*, 2016).

The T3Es of *Pab* or *Pag* could be divided into three groups:

- HsvB and HsvG are paralogous T3Es that mimic host‐specific transcriptional activators on beet and gypsophila, respectively, and determine pathovar specificity (Nissan *et al.*, 2006, 2012; Valinsky *et al.*, 1998). Both are present in each pathovar and are functional only in the corresponding host; HsvG is required for gypsophila infection and HsvB for beet infection. Replacement of the HsvG promoter with a stronger promoter, equivalent to *hrpJp*, caused an increase in gall size of up to three times, suggesting that HsvG, and presumably HsvB, may interfere with the plant hormone balance leading to gall development (Nissan *et al.*, 2005).
- PthG and PseB are exclusively present as active T3Es in *Pag* and *Pab*, respectively (Nissan *et al.*, 2018). PthG supports disease development in gypsophila and triggers HR on multiple beet species (Ezra *et al.*, 2000, 2004). In *Pab*, PthG is truncated and nonfunctional, allowing *Pab* to infect beet. Similarly, *Pag* mutated in the PthG gene infects beet as well as gypsophila (Ezra *et al.*, 2000). PseB is a novel T3E from *Pab* that is exclusively present in this pathovar with as yet unknown function (Nissan *et al.*, 2018).
- The remaining T3Es are common to other Gram‐negative phytopathogenic bacteria and may contribute to gall development by diverse mechanisms. To the best of our knowledge, HsvB, HsvG, PseB and PthG have not been reported as functional T3Es in any other pathogenic bacteria and presumably could have evolved through a pathoadaptive evolution (Sokurenko *et al.*, 1999). In contrast, the remaining effectors are shared with other phytopathogenic bacteria and presumably have been acquired by horizontal gene transfer (HGT).

The contribution of each T3E to virulence was quantitatively assessed by comparing the gall’s fresh weight incited by the wild‐type strain with that incited by its mutant (Nissan *et al.*, 2018). The highest contribution was provided by HsvG on gypsophila and HsvB on beet as a mutation in the corresponding T3Es caused a >95% reduction in gall formation in gypsophila and beet, respectively. Mutants in either PthG in *Pag* or PseB in *Pab* also caused a significant reduction in gall size but considerably lower than the former two effectors (Nissan *et al.*, 2018).

This study was undertaken to determine the minimal combination of T3Es in *Pag* or *Pab* that is sufficient to elicit galls on either gypsophila or beet. The adopted strategy was to convert nonpathogenic Gram‐negative bacteria into gall‐forming pathogens on either gypsophila or beet by transformation of T3Es taken from the two *Pa* pathovars. Initially, the nonpathogenic bacteria were provided with the capability to translocate T3Es into plant cells via transformation of pHIR11, a cosmid harbouring a plant‐adapted T3SS (Huang *et al.*, 1988), followed by transformation of T3Es from *Pag* or *Pab*. The T3Es HsvG, HsvB, PthG and PseB were selected for this study because they presumably were evolved by pathoadaptive evolution and apparently were instrumental in the emergence of *Pag* and *Pab* as new pathogens.

The present communication demonstrates that transformation of HsvG and PthG or HsvB and PseB converts nonpathogenic bacteria into host‐specific gall‐forming pathogens on gypsophila and beet, respectively, with the exception of *E. coli* strains that could support gall development only on gypsophila. Moreover, transformation of each of the above two T3E pairs into three major T3SS‐dependent phytopathogenic bacteria allowed them to expand their host range and incite galls on gypsophila or beet in a host‐specific manner without modifying their own characteristic symptoms on the natural hosts.

The bacterial strains, plasmids and a cosmid used in this study are described in Table S1. Wild‐type strains of *Pag* and *Pab*, as well as other phytopathogenic bacteria employed in this study, were grown in Luria‐Bertani (LB) agar at 28 °C, whereas *E. coli* strains were cultured on the same medium at 37 °C. Antibiotics were used at the following concentrations (µg/mL): ampicillin (Amp), 150; kanamycin (Km), 50; rifampicin (Rif), 150; spectinomycin (Spec), 50; tetracycline (Tc), 15.

Pathogenicity tests on cuttings of *Gypsophila paniculata* 'Golan' (Danziger Ltd, Bet Dagan, Israel) were essentially performed according to Lichter *et al*. (1995) as described by Nissan *et al.* (2018). After removal of an approximately 2 mm section from the bottom of the stem, the cuttings (ten for each treatment) were inoculated by dipping into a bacterial suspension of 10^6^ cells/mL for 30 min and placed in vermiculite‐filled trays for symptom visualization. The glasshouse temperature was maintained at 22–25 °C and high humidity was generated by computer‐controlled mist sprinklers that were activated every 20 min for 10 s. Pathogenicity was scored 10–15 days after inoculation. The degree of virulence was determined by removal of the galls from the infected cuttings and measurement of their fresh weight. Pathogenicity tests on table beet cubes were performed according to Ezra *et al*. (2000). Whole matured beets (*Beta* *vulgaris* 'Egyptian Red Beet') were soaked in 1% hypochlorite for 10 min following by two washings in sterile water. They were then cut into approximately 0.5 × 0.7 × 0.7 cm under sterile conditions and placed on sterile 1.5% water agar in a Petri dish. Inoculation was carried out with a culture grown overnight on LB agar by puncturing the top of the cube and inserting the bacteria with a sterile toothpick (five cubes per each treatment). Virulence was scored following incubation of the cubes for 5 days at 28 °C. Pathogenicity experiments were conducted in a quarantine greenhouse. Pathogenicity of the phytopathogenic bacteria on their natural hosts, namely, *Erwinia amylovora* (*Ea*) on pear blossom clusters, *Dickeya solani* (*Ds*) on potato tubers and *Xanthomonas campestris* pv. *campestris* (*Xcc*) on cabbage seedlings, were performed according to Kleitman *et al*. (2005), Schaad *et al. *(2001) and Tsror (Lahkim) *et al*. (2013), respectively.

Isolation of DNA from *Pa* or *E. coli* strains, cloning, ligation, transformation and other DNA manipulations were performed according to standard procedures (Ausubel *et al.*, 1995) or as recommended by the supplier. The cloning vectors used in this study are listed in Table S1. Transfer of T3Es cloned in *E. coli* DH5α into nonpathogenic or pathogenic bacterial strains was performed by triparental mating with the *E. coli* helper plasmid pRK2073 (Spt^r^) essentially as described elsewhere (Ditta *et al.*, 1980; Manulis *et al.*, 1998). The recipient and the helper bacteria were mixed on LB agar plates, incubated at 28 °C overnight and then plated on LB agar with appropriate antibiotics. Curing of the desired bacterial strain from transconjugants was carried out by subculturing in the absence of antibiotic selection as previously described (Manulis *et al.*, 1998).

The nonpathogenic bacterial strain *Pseudomonas fluorescens* 55 (*Pf*) harbouring the cosmid pHIR11 that encodes a functional T3SS from *P. syringae* (Huang *et al.*, 1988) as well as the nonpathogenic strains of *Pa*, *Pa *3‐1 and *Pa *98, and two *E. coli* strains (Table S1) were employed for transformation into gall‐forming pathogens. The *E. coli* strains included a shiga toxin mutant of the enterohemorrhagic *E. coli* (EHEC) designated as TUV93‐0 and *E. coli* DH5α (Table S1). To endow the nonpathogenic bacterial strains with the capability of translocating T3Es into plant cells, they were initially transformed with pHIR11 to obtain *Pa *3‐1 (pHIR11) *Pa *98 (pHIR11), EHEC TUV93‐0 (pHIR11) and *E. coli* DH5α (pHIR11).

The T3E genes *hsvG* and *hsvB* were separately cloned into pQE70 (Amp^r^), whereas *pthG* and *pseB* were separately cloned into pVSP61 (Kan^r^) (Table S1). The cloned plasmids were transformed into *E. coli* DH5α and mobilized by triparental mating in various combinations into *Pf*, *Pa *3‐1 (pHIR11), *Pa *98 (pHIR11), *E. coli* shiga mutant TUV93‐0 (pHIR11), *E. coli* DH5α (pHIR11) and the phytopathogenic strains *Ea*, *Ds* and *Xcc* (Table S1). The presence of the transformed effectors was validated by PCR using primers given in Table S2.

Results presented in Table 1 and Fig. 1 indicate that nonpathogenic bacterial strains harbouring *hsvG* and *pthG* elicited galls on gypsophila whereas nonpathogenic bacterial strains harbouring *hsvB* and *pseB* elicited galls on beet with the exception of the *E. coli* strains. Bacterial strains containing *pthG* generally elicited HR on beet (Ezra *et al.*, 2000). EHEC TUV93‐0 (pHIR11) triggered HR on beet, whereas no symptoms could be observed with *E. coli* DH5α (pHIR11) (Table 1). Interestingly, the HR response could not be observed with EHEC TUV93‐0 lacking pHIR11, suggesting that the HR might be caused by a translocated T3E of EHEC TUV93‐0, which is absent in *E. coli* DH5α. The inability of the *E. coli* strains harbouring *hsvB* and *pseB* to incite galls on beet is not yet understood and may only be hypothesized. EHEC TUV93‐0 (a derivative of *E. coli* 0157:H70) is a shiga toxin mutant of human and animal pathogen that survives well on plants (Wright *et al.*, 2013) and harbours T3Es for virulence (Tobe *et al.*, 2006). In contrast, *E*. *coli* DH5α is a genetically engineered bacterial strain used to facilitate cloning and lacks any T3Es (Taylor *et al.*, 1993). The HR of beet to EHEC TUV93‐0 (pHIR11) could prevent gall formation as previously described for PthG of *Pag* (Ezra *et al.*, 2000). Additionally, a minimal degree of endophytic bacterial growth might be considered a prerequisite for translocation of T3Es into a plant’s cell. The two *E. coli* strains most likely differ in their degree of endophytic growth; while EHEC TUV93‐0 is adapted for plant colonization, *E*. *coli* DH5α is not. Nevertheless, the nutrients released from wounded gypsophila cuttings could be sufficient for translocation of T3Es and formation of small galls (Fig. 1).

**Table 1:** Transformation of nonpathogenic bacteria into host‐specific gall‐inciting pathogens on beet or gypsophila

Bacterial strain*	Gall formation on beet†	Gall formation on gypsophila†
*Pseudomonas fluorescens* 55 cloned with pHIR11 (*Pf*)	−	−
*Pf* (pQE70, pVSP61)	−	−
*Pf* (*hsvG*)	−	−
*Pf* (*pthG*)	HR	−
*Pf* (*hsvG* + *pthG*)	HR	+
*Pf* (*hsvB*)	−	−
*Pf* (*pseB*)	−	−
*Pf* (*hsvB* + *pseB*)	+	−
*Pantoea agglomerans* 3‐1(*Pa *3‐1), wild type	−	−
*Pa *3‐1 (pHIR11, pQE70, pVSP61)	−	−
*Pa *3‐1 (pHIR11 + *hsvG*)	−	−
*Pa *3‐1 (pHIR11 + *pthG*)	HR	−
*Pa *3‐1 (pHIR11 + *hsvG* + *pthG*)	HR	+
*Pa *3‐1 (pHIR11 + *hsvB*)	−	−
*Pa *3‐1 (pHIR11 + *pseB*)	−	−
*Pa *3‐1 (pHIR11 + *hsvB* + *pseB*)	+	−
*Pantoea agglomerans* BRT98 (*Pa *98), wild type	−	−
*Pa *98 (pHIR11, pQE70, pVSP61)	−	−
*Pa *98 (pHIR11 + *hsvG*)	−	−
*Pa *98 (pHIR11 + *pthG*)	HR	−
*Pa *98 (pHIR11 + *hsvG* + *pthG*)	HR	+
*Pa *98 (pHIR11 + *hsvB*)	−	−
*Pa *98 (pHIR11 + *pseB*)	−	−
*Pa *98 (pHIR11 + *hsvB*+ *pseB*)	+	−
Enterohemorrhagic *Escherichia coli* (EHEC TUV93‐0, a shiga toxin mutant)	−	−
EHEC TUV93‐0 (pHIR11)	HR	−
EHEC TUV93‐0 (pHIR11, pQE70, pVSP61)	HR	−
EHEC (pHIR11 + *hsvG* + *pthG*)	HR	+
EHEC (pHIR11 + *hsvB* + *pseB*)	HR	−
*E*. *coli* DH5α (*Ec*)	−	−
*Ec* (pHIR11)	−	−
*Ec* (pHIR11, pQE70, pVSP61)	−	−
*Ec* (pHIR11 + *hsvG* + *pthG*)	−	+
*Ec* (pHIR11 + *hsvB* + *pseB*)	−	−

* pHIR11 is a cosmid clone harbouring a functional T3SS of *Pseudomonas syringae* pv. *syringae*. *hsvG* or *pseB* effector genes were cloned in the vector pQE70, whereas *pthG* or *hsvB* effector genes were cloned in the vector pVSP61 prior to transformation into the nonpathogenic bacterial strains.

† −, no symptoms; +, gall formation; HR, hypersensitive response elicitation.

**Figure 1:**
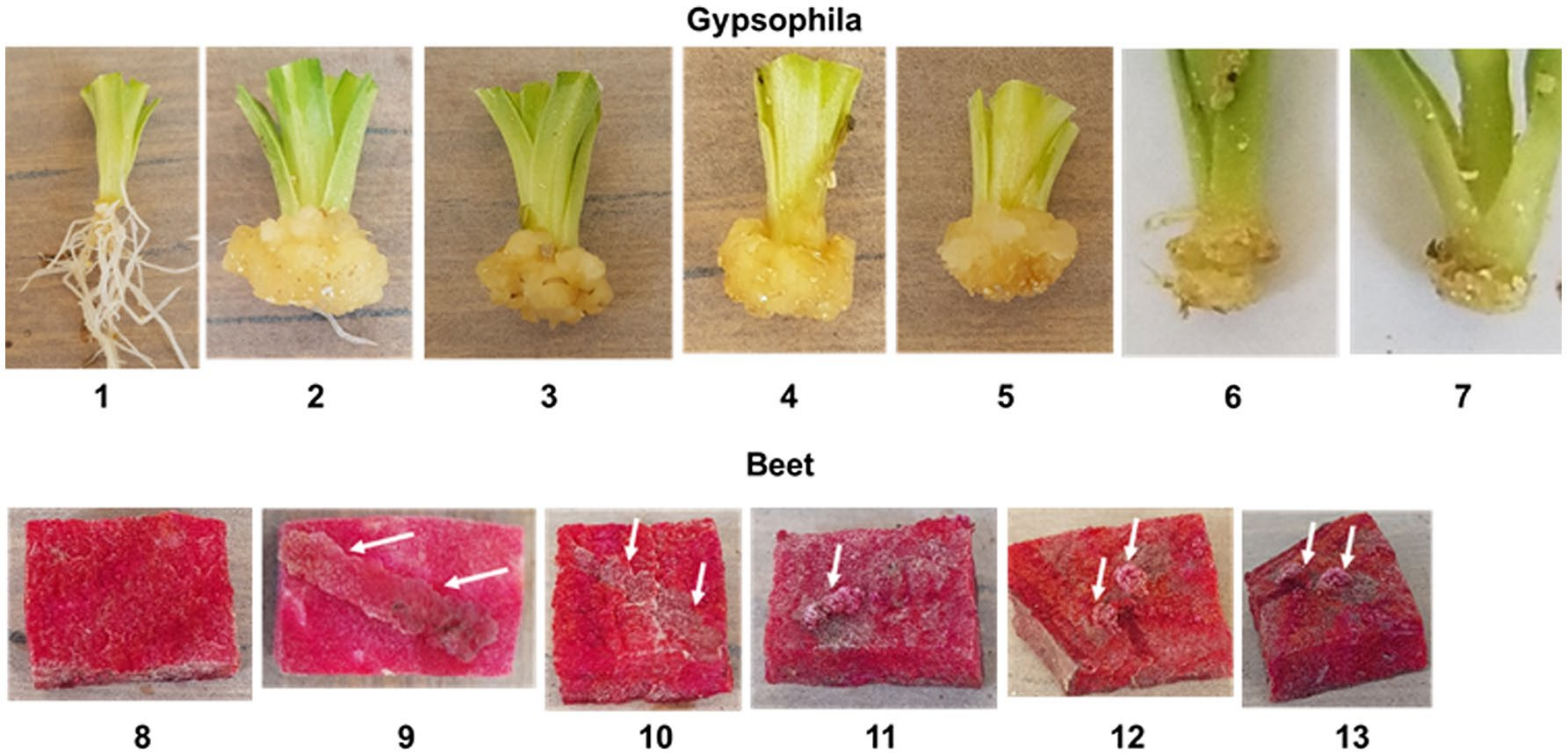
Pathogenicity tests on gypsophila cuttings and beet roots. Gypsophila cuttings: 1, water control; 2, *Pantoea agglomerans * pv. *gypsophilae* 824‐1; 3, *Pseudomonas fluorescens* 55 (*Pf*) (*hsvG* + *pthG*); 4, *P. agglomerans* (*Pa*) 3‐1 (*hsvG* + *pthG*); 5, *Erwinia amylovora* (*hsvG + pthG*); 6, *Escherichia coli* (EHEC TUV93‐0) (hsvG + pthG); 7, *E. coli* DH5α (*hsvG* + *pthG*). Beet roots: 8, water control; 9, *P.* *agglomerans* pv. *betae* 4188 (arrows point to gall); 10, *Pf* (*hsvG* + *pthG*) showing hypersensitive response symptom (indicated by arrows); 11, *Pf* (*hsvB* + *pseB*) (gall); 12, *Pa* 3‐1 (*hsvB* + *pseB*) (gall); 13, *E. amylovora* (*hsvB* + *pseB*) (gall). Photos of gypsophila cuttings were taken 14 days after inoculation and for beet roots 5 days after inoculation.

The galls’ fresh weight in gypsophila inoculated by the reconstructed pathogenic strains, *Pf*, *Pa *3‐1 or *Pa *98, were generally smaller by up to 30% than those produced by the wild type (*Pag* 824‐1). Average gall size for *Pag *824‐1 was 215 ± 20 mg and for *Pf*, *Pa *3‐1 or *Pa *98 containing *hsvG* and *pthG* was 156 to 165 ± 20 mg. The latter observation might suggest that the remaining T3Es present in *Pag *824‐1 contribute to maximal gall size.

The ability of the two pairs of T3Es to transform nonpathogenic bacteria into host‐specific gall‐forming pathogens on beet or gypsophila prompted us to examine whether these plasmid‐cloned effectors can also transform T3SS‐dependent phytopathogenic bacteria into host‐specific pathogens on the same two hosts. To resolve this question, three phytopathogenic bacteria, namely, *Ea, Ds* and *Xcc* (Table S1), were transformed by three‐parental mating with HsvG, HsvB, PthG and PseB in various combinations as described above for the nonpathogenic strains. Pathogenicity tests performed under quarantine conditions indicated that strains of the three tested bacteria harbouring the HsvG‐PthG pair and the HsvB‐PseB pair incited gall formation on gypsophila and beet, respectively (Table 2 and Fig. 1). Pathogenicity tests with the transformed pathogens listed in Table 2 were also conducted on their natural hosts, namely pear for *Ea*, potato for *Ds* and cabbage for *Xcc*, as indicated above. The characteristic symptoms for each of these pathogens on their natural hosts were preserved without detecting gall appearance (results not shown). These results indicate that plasmid‐cloned T3Es can be maintained by bacterial pathogens without modifying their activity on their compatible hosts.

**Table 2:** Transformation of T3SS‐dependent phytopathogenic bacteria into host‐specific gall‐inciting pathogens on beet or gypsophila

Bacterial strain*	Gall formation on beet†	Gall formation on gypsophila†
*Erwinia amylovora* 238 (*Ea*), wild type	−	−
*Ea* (pQE70, pVSP61)	−	−
*Ea* (*hsvG*)	−	−
*Ea* (*pthG*)	HR	−
*Ea* (*hsvG* + *pthG*)	HR	**+**
*Ea* (*hsvB*)	−	−
*Ea* (*pseB*)	−	−
*Ea* (*hsvB* + *pseB*)	**+**	−
*Dickeya solani* 3228 (*Ds*), wild type	−	−
*Ds* (pQE70, pVSP61)	−	−
*Ds* (*hsvG*)	−	−
*Ds* (*pthG*)	HR	−
*Ds* (*hsvG* + *pthG*)	HR	**+**
*Ds* (*hsvB*)	−	−
*Ds* (*pseB*)	−	−
*Ds* (*hsvB* + *pseB*)	**+**	−
*Xanthomonas campestris* pv*. campestris* R105 (*Xcc*) wild type	−	−
*Xcc* (pQE70, pVSP61)	−	−
*Xcc* (*hsvG*)	−	−
*Xcc* (*pthG*)	HR	−
*Xcc* (*hsvG* + *pthG*)	HR	**+**
*Xcc* (*hsvB*)	−	−
*Xcc* (*pseB*)	−	−
*Xcc* (*hsvB* + *pseB*)	**+**	−

* *hsvG* or *pseB* effector genes were cloned into the vector pQE70, whereas *pthG* or *hsvB* effector genes were cloned into the vector pVSP61 prior to transformation into the phytopathogenic bacteria.

† −, no symptoms; +, gall formation; HR, hypersensitive response elicitation.

A previous attempt to deal with a minimal repertoire of T3Es required for plant disease development was carried out with *P. syringae* pv. *tomato (Pst*) strain DC3000 in which eight out of 28 T3Es were sufficient to confer near wild‐type bacterial growth and disease symptoms in *Nicotiana benthamiana* plants (Cunnac *et al.*, 2011). The significant difference in the numbers of minimal effectors between *Pag* or *Pab* and *Pst* DC3000 could be assigned to the evolutionary stage of the two pathogens as well as to the nature of their T3Es. The chromosomal location of the T3SS in *Pst* DC3000 and its effectors, as well as the substantial number of T3Es that have been accumulated during the co‐evolutionary arms race between host and pathogen (Starvinides *et al.*, 2008), are indicative of a long evolutionary period. In contrast, as described earlier, *Pag* and *Pab* are newly evolved pathogens that harbour only a plasmid‐borne T3SS and a considerably smaller number of plasmid‐borne T3Es. Therefore, the number of minimal indispensable effectors that are accumulated during the evolution of *Pst* DC3000 is expected to be significantly higher than in *Pantoea*.

The transformation of nonpathogenic *Pa* into a gall‐forming pathogen is essentially dependent on the emergence of HsvG and HsvB as a unique new class of host‐specific transcriptional activators (Nissan *et al.*, 2006, 2012). T3Es acting as host transcriptional activators provide an effective strategy to manipulate plant gene expression (Buttner, 2016). Type III effector proteins, which can directly be imported into the nucleus and bind to either DNA or to components of the plant transcription machinery, have been previously exemplified by the transcription activator‐like (TAL) effectors that can efficiently modify cellular processes (Boch *et al.*, 2014; Buttner, 2016). HsvG and HsvB are structurally different from TAL effectors (Buttner, 2016; Canonne and Rivas, 2012). They can be localized to the plant cell nucleus of host and non‐host plants, harbour nuclear localization signals, which are required for *Pa* pathogenicity as well as helix‐turn‐helix domain containing a DNA binding motif and possibly responsible for additional functions (Nissan *et al*, 2006, 2012; Weinthal *et al.*, 2011). The activation domain of HsvG has two nearly direct repeats (71 and 74 amino acids) whereas that of HsvB has only one repeat. Exchanging the activation domain of HsvG and HsvB resulted in a switch in host specificity (Nissan *et al.*, 2006).

A candidate target gene of HsvG in gypsophila is *HSVGT*, which encodes a predicted acidic protein harbouring characteristic conserved motifs of eukaryotic transcription factors (Nissan *et al.*, 2012). *HSVGT* is transcriptionally induced *in planta* by *Pag* and is dependent on intact *hsvG*. It was confirmed as a direct target of HsvG by gel‐shift assays showing that HsvG binds to the *HSVGT* promoter. It is possible that the HsvG‐mediated activation of the putative transcription factor HSVGT results in the activation of additional plant genes that lead to gall development. Further studies on the interactions between HsvG and HsvB and the transcriptomes of their specific host plants should elucidate the mechanisms for hyperplasia and hypertrophy leading to gall formation.

The dominant contribution of HsvG or HsvB as novel transcriptional activators that also determine pathovar specificity make them indispensable for the emergence of gall‐forming *Pantoea*. The role of the two additional T3Es (i.e. PthG and PseB) that were also presumably evolved by pathoadaptation remains to be clarified as well as whether they can be replaced by any of the remaining T3Es.

## Supporting information


**Table S1** Bacterial strains, cosmid and plasmids used in this study.

**Table S2** Primers used for confirmation of type III effectors employed in this study.
