# Supplementary material for: Two Pantoea agglomerans type III effectors can transform nonpathogenic and phytopathogenic bacteria into host‐specific gall‐forming pathogens
Source: Mol Plant Pathol. 2019 Aug 1;20(11):1582–7. doi: 10.1111/mpp.12860 (PMC6804341; doi:10.1111/mpp.12860)
Supplement: Supplementary file 1 — Table S1 Bacterial strains, cosmid and plasmids used in this study. [file MPP-20-1582-s001.docx]

**Table S1.** Bacterial strains, cosmids and plasmids used in this study

| **Strain or plasmid** | **Relevant characteristics** | **Reference or source** |
| --- | --- | --- |
| ***Escherichia coli*** |  |  |
| DH5α (*Ec*) | *lacZ*ΔM15 Δ(*lacZ*YA-*arg*F)U169 *gyr*A96 | Invitrogen, California, USA |
| *Ec* /pHIR11 | Tc^R^, DH5α harboring the cosmid pHIR11. | This study |
| *Ec* (pHIR11, pQE70, pVSP61) | Tc^R^, Km^R^, Amp^R^, DH5α harboring the cosmid pHIR11 and the vectors pQE70 and pVSP61. | This study |
| *Ec* (pHIR11+*hsvG* + *pthG*) | Tc^R^, Amp^R^, Km^R^, DH5α harboring pHIR11 and the plasmids pVSP-*pthG* and pQE70-*hsvG*. | This study |
| *Ec* (pHIR11+*hsvB* + *pseB*) | Tc^R^, Amp^R^, Km^R^, DH5α harboring pHIR11 and the plasmids pVSP-*hsvB* and pQE70-*pseB*. | This study |
| Enterohemorrhagic *E. coli* (EHEC)  EDL933 slt1-Slt2 | a shiga toxin mutant (EHEC TUV93-0) | Ilan Rosenshine,  Hebrew University, Jerusalem, Israel |
| EHEC TUV93-0 (pHIR11) | Tc^R^, EHEC harboring the cosmid pHIR11. | This study |
| EHEC TUV93-0 (pHIR11, pQE70, pVSP61) | Tc^R^, Km^R^, Amp^R^, EHEC harboring the cosmid pHIR11 and the vectors pQE70 and pVSP61. | This study |
| EHEC (pHIR11+*hsvG* + *pthG*) | Tc^R^, Amp^R^, Km^R^, EHEC harboring pHIR11 and the plasmids pVSP-*pthG* and pQE70-*hsvG*. | This study |
| EHEC (pHIR11+*hsvB* + *pseB*) | Tc^R^, Amp^R^, Km^R^, EHEC harboring pHIR11 and the plasmids pVSP-*hsvB* and pQE70-*pseB*. | This study |
| ***Pantoea agglomerans*** |  |  |
| *Pag*824-1 | Rif^R^, *Pantoea agglomerans* pv. *gypsophilae* (*Pag*), wild-type pathogenic strain, gypsophila pathovar. | [Manulis *et al*. (1991](http://onlinelibrary.wiley.com/doi/10.1111/j.1365-2958.2006.05301.x/full#b7)) |
| *Pab*4188 | Rif^R^, *Pantoea agglomerans* pv. *betae* (*Pab*) wild-type pathogenic strain, beet pathovar. | [Burr *et al*. (1991](http://onlinelibrary.wiley.com/doi/10.1111/j.1365-2958.2006.05301.x/full#b6)) |
| *Pa*3-1 | Rif^R^, *Pantoea agglomerans* 3-1, wild-type nonpathogenic strain. | [Manulis *et al*. (1991](http://onlinelibrary.wiley.com/doi/10.1111/j.1365-2958.2006.05301.x/full#b7)) |
| *Pa*3-1/pHIR11/ pQE70/ pVSP61 | Rif^R^, Tc^R^, Km^R^, Amp^R^, *Pa*3-1 harboring the cosmid pHIR11 and the vectors pQE70 and pVSP61. | This study |
| *Pa*3-1/pHIR11 | Rif^R^, Tc^R^, *Pa*3-1 harboring the cosmid pHIR11. | This study |
| *Pa*3-1/pHIR11/pVSP-*pthG* | Rif^R^, Tc^R^, Km^R^, *Pa*3-1 harboring pHIR11 and the plasmid pVSP-*pthG*. | This study |
| *Pa*3-1/pHIR11/pQE70-*hsvG* | Rif^R^, Tc^R^, Amp^R^, *Pa*3-1 harboring pHIR11 and the plasmid pQE70-*hsvG*. | This study |
| *Pa*3-1/pHIR11/pVSP-*pthG*/pQE70-*hsvG* | Rif^R^, Tc^R^, Amp^R^, Km^R^, *Pa*3-1 harboring pHIR11 and the plasmids pVSP-*pthG* and pQE70-*hsvG*. | This study |
| *Pa*3-1/pHIR11/pVSP-*hsvB* | Rif^R^, Tc^R^, Km^R^, *Pa*3-1 harboring pHIR11 and the plasmid pVSP-*hsv*B. | This study |
| *Pa*3-1/ pHIR11/pQE70-*pseB* | Rif^R^, Tc^R^, Amp^R^, *Pa*3-1 harboring pHIR11 and the plasmid pQE70-*pseB*. | This study |
| *Pa*3-1/pHIR11/pVSP-*hsvB*/pQE70-*pseB* | Rif^R^, Tc^R^, Amp^R^, Km^R^, *Pa*3-1 harboring pHIR11 and the plasmids pVSP-*hsvB* and pQE70-*pseB*. | This study |
| BRT98 (*Pa*98) | Rif^R^, *Pantoea agglomerans*, wild-type nonpathogenic strain. | Lindow Laboratory |
| *Pa*98/pHIR11/ pQE70/ pVSP61 | Rif^R^, Tc^R^, Km^R^, Amp^R^, *Pa*98 harboring the cosmid pHIR11 and the vectors pQE70 and pVSP61 | This study |
| *Pa*98/pHIR11 | Rif^R^, Tc^R^, *Pa*98 harboring the cosmid pHIR11 | This study |
| *Pa*98/pHIR11/pVSP-*pthG* | Rif^R^, Tc^R^, Km^R^, *Pa*98 harboring pHIR11 and the plasmid pVSP-*pthG* | This study |
| *Pa*98/pHIR11/pQ70-*hsvG* | Rif^R^, Tc^R^, Amp^R^, *Pa*98 harboring pHIR11 and the plasmid pQE70-*hsvG*. | This study |
| *Pa*98/pHIR11/pVSP-*pthG*/pQE70-*hsvG* | Rif^R^, Tc^R^, Amp^R^, Km^R^, *Pa*98 harboring pHIR11 and the plasmids pVSP-*pthG* and pQE70-*hsvG*. | This study |
| *Pa*98/pHIR11/pVSP-*hsvB* | Rif^R^, Tc^R^, Km^R^, *Pa*98 harboring pHIR11 and the plasmid pVSP-*hsv*B. | This study |
| *Pa*98/ pHIR11/pQE70-*pseB* | Rif^R^, Tc^R^, Amp^R^, *Pa*98 harboring pHIR11 and the plasmid pQE70-*pseB*. | This study |
| *Pa*98/pHIR11/pVSP-*hsvB*/pQE70-*pseB* | Rif^R^, Tc^R^, Amp^R^, Km^R^, *Pa*98 harboring pHIR11 and the plasmids pVSP-*hsvB* and pQE70-*pseB*. | This study |
| **Pseudomonas fluorescens 55**/ pHIR11 (*Pf*) | Tc^R^, Pf, a nonpathogenic strain harboring the cosmid pHIR11. | Huang *et al*. (1998). |
| Pf / pHIR11/pQE70/pVSP61 | Tc^R^, Amp^R^, Km^R^, *Pf* harboring the vectors pQE70 and pVSP61. | This study |
| *Pf*/ pHIR11/pVSP-*pthG* | Tc^R^, Km^R^, *Pf* harboring pHIR11 and the plasmid pVSP-*pthG* | This study |
| *Pf*/ pHIR11/pQ70-*hsvG* | Tc^R^, Amp^R^, *Pf* harboring pHIR11 and the plasmid pQE70-*hsvG* | This study |
| *Pf*/ pHIR11/pVSP-*pthG*/pQE70-*hsvG* | Tc^R^, Amp^R^, Km^R^, *Pf* harboring pHIR11 and the plasmids pVSP-*pthG* and pQE70-*hsvG*. | This study |
| Pf/ pHIR11/pVSP-*hsvB* | Tc^R^, Km^R^, *Pf* harboring pHIR11 and the plasmid pVSP-*hsv*B. | This study |
| Pf/ pHIR11/pQE70-*pseB* | Tc^R^, Amp^R^, *Pf* harboring pHIR11 and the plasmid pQE70-*pseB*. | This study |
| Pf/ pHIR11/pVSP-*hsvB*/pQE70*-pseB* | Tc^R^, Amp^R^, Km^R^, *Pf* harboring pHIR11 and the plasmids pVSP-*hsvB* and pQE70-*pseB*. | This study |
| ***Erwinia amylovora* 238** (*Ea*) | Rif^R^, wild type, pathogenic strain. | Manulis Laboratory |
| *Ea*/pQE70/pVSP61 | Rif^R^, Amp^R^, Km^R^, *Ea* harboring the vectors pQE70 and pVSP61. | This study |
| *Ea*/pVSP-*pthG* | Rif^R^, Km^R^, *Ea* harboring pVSP-*pthG*. | This study |
| *Ea*/ pQE70-*hsvG* | Rif^R^, Amp^R^, *Ea* harboring pQE70-*hsvG*. |  |
| *Ea* /pVSP-*pthG/*pQE70-*hsvG* | Rif^R^, Amp^R^, Km^R^, *Ea* harboring the plasmids pVSP-*pthG* and pQE70-*hsvG*. | This study |
| *Ea*/pVSP-*hsvB* | Rif^R^, Km^R^, *Ea* harboring pVSP-*hsvB*. | This study |
| *Ea*/pQE70- *pseB* | Rif^R^, Amp^R^, *Ea* harboring pQE70- *pseB*. | This study |
| *Ea* /pVSP-*hsvB*/pQE70-*pseB* | Rif^R^, Amp^R^, Km^R^, *Ea* harboring the plasmids pVSP-*hsvB* and pQE70-*pseB*. | This study |
| ***Dickeya solani* 3228** (*Ds*) | Rif^R^, wild type, pathogenic strain. | Manulis laboratory |
| *Ds*/pQE70/pVSP61 | Rif^R^, Amp^R^, Km^R^, *Ds* harboring the vectors pQE70 and pVSP61. | This study |
| *Ds* /pVSP-*pthG* | Rif^R^, Km^R^, *Ds* harboring pVSP-*pthG*. | This study |
| *Ds* / pQE70-*hsvG* | Rif^R^, Amp^R^, *Ds* harboring pQE70-*hsvG*. |  |
| *Ds* /pVSP-*pthG/*pQE70-*hsvG* | Rif^R^, Amp^R^, Km^R^, *Ds* harboring the plasmids pVSP-*pthG* and pQE70-*hsvG*. | This study |
| *Ds* /pVSP-*hsvB* | Rif^R^, Km^R^, *Ds* harboring pVSP-*hsvB*. | This study |
| *Ds* /pQE70- *pseB* | Rif^R^, Amp^R^, *Ds* harboring pQE70- *pseB*. | This study |
| *Ds* /pVSP-*hsvB*/pQE70-*pseB* | Rif^R^, Amp^R^, Km^R^, *Ds* harboring the plasmids pVSP-*hsvB* and pQE70-*pseB*. | This study |
| ***X. campestris* pv. *campestris*** **R105** (*Xcc*) | Rif^R^, wild type, pathogenic strain. | Manulis Laboratory |
| *Xcc* /pQE70/pVSP61 | Rif^R^, Amp^R^, Km^R^, *Xcc* harboring the vectors pQE70 and pVSP61. | This study |
| *Xcc* /pVSP-*pthG* | Rif^R^, Km^R^, *Xcc* harboring pVSP-*pthG*. | This study |
| *Xcc* / pQE70-*hsvG* | Rif^R^, Amp^R^, *Xcc* harboring pQE70-*hsvG*. |  |
| *Xcc* /pVSP-*pthG/*pQE70-*hsvG* | Rif^R^, Amp^R^, Km^R^, *Xcc* harboring the plasmids pVSP-*pthG* and pQE70-*hsvG*. | This study |
| *Xcc* /pVSP-*hsvB* | Rif^R^, Km^R^, *Xcc* harboring pVSP-*hsvB*. | This study |
| *Xcc* /pQE70- *pseB* | Rif^R^, Amp^R^, *Xcc* harboring pQE70- *pseB*. | This study |
| *Xcc* /pVSP-*hsvB*/pQE70-*pseB* | Rif^R^, Amp^R^, Km^R^, *Xcc* harboring the plasmids pVSP-*hsvB* and pQE70-*pseB*. | This study |
| **Cosmid** |  |  |
| pHIR11 | Tc^R^, encoded a functional T3SS. | Huang *et al*. (1988) |
| **Plasmids** |  |  |
| pVSP61 | Km^R^, pVS1 replicon | Loper and Lindow (1994) |
| pQE70 | Ap^R^, His-tag expression vector. | Qaigen, San Francisco, USA |
| pQE70-*hsvG* | Ap^R^, PCR-amplified *hsvG* coding sequence cloned into pQE70 as a *Sph*I/*Bam*HI fragment. | Nissan *et* *al*. (2006). |
| pQE70-*pseB* | Ap^R^, PCR-amplified *pseB* coding sequence cloned into pQE70 as a *Sph*I/*Bam*HI fragment. | Nissan *et* *al*. (2018). |
| pVSP-*pthG* | Kn^R^, PCR-amplified *pthG* coding sequence cloned into pVSP61 as a *HindIII*/*BamHI* fragment. | Ezra *et* *al*. (2000). |
| pVSP-*hsvB* | Km^R^, PCR-amplified *hsvB* coding sequence cloned into pVSP61 as a *HindIII*/*BamHI* fragment | Nissan *et* *al*. (2006). |

**REFERENCE**

Loper, J. E., and Lindow, S. E. 1994. A biological sensor for iron available to bacteria in their habitats on plant surfaces. Appl. Environ. Microbiol*.* 60: 1934-1941
