# Supplementary material for: Two Pantoea agglomerans type III effectors can transform nonpathogenic and phytopathogenic bacteria into host‐specific gall‐forming pathogens
Source: Mol Plant Pathol. 2019 Aug 1;20(11):1582–7. doi: 10.1111/mpp.12860 (PMC6804341; doi:10.1111/mpp.12860)
Supplement: Supplementary file 2 — Table S2 Primers used for confirmation of type III effectors employed in this study. [file MPP-20-1582-s002.docx]

**Table S2:** Primers used for confirmation of type III effectors in this study**.**

| **Primer name** | **Sequence (5’-3’)** | **Used For** |
| --- | --- | --- |
| vg-d | GCCCAGGCAGGGACATTTAT | Confirmation of the presence of *hsvG* |
| vg-r | GGAAAGCGGGTCTGAAGTGT | “ |
| vb-d | GAAGCGCAGATTAAGGCAGC | Confirmation of the presence of *hsvB* |
| vb-r | GCCTGACCAGTCTGAGCC | “ |
| pt-d | GTTTCAACGTTACGGGTGCC | Confirmation of the presence of *pthG* |
| pt-r | CCGGTGCTAATAGCTTGCCT | “ |
| ps-d | CATCAGCCCATGAAACCCGA | Confirmation of the presence of *pseB* |
| ps-r | TCTTGAACGTGCTGAGTGTT | “ |
